# Lemborexant, a Dual Orexin Receptor Antagonist, ameliorates Tau‐mediated Sleep Loss and Neurodegeneration

**DOI:** 10.1002/alz.089253

**Published:** 2025-01-03

**Authors:** Samira Parhizkar, Wei Chen, Xin Bao, Grace Gent, Nicholas Rensing, Eric Tycksen, Emily Franke, Melissa Manis, Megan E. Bosch, Choonghee Lee, Javier R. Serrano, Eric Landsness, Michael Wong, David M. Holtzman

**Affiliations:** ^1^ Washington University School of Medicine, St. Louis, MO USA; ^2^ Hope Center for Neurological Disorders, Washington University School of Medicine, St. Louis, MO USA; ^3^ Washington University School of Medicine in St Louis, St. Louis, MO USA; ^4^ Knight Alzheimer Disease Research Center, St. Louis, MO USA

## Abstract

**Background:**

Sleep disturbances are associated with the pathogenesis of neurodegenerative diseases including Alzheimer’s disease (AD) and primary tauopathies. We have previously shown that APOE4, the strongest genetic risk factor for AD, directly influences the severity of key pathological hallmarks of neurodegeneration including tau deposition, microglial reactivity and brain atrophy. Sleep loss influences tau accumulation and microglial reactivity in both mice and humans, suggesting that sleep loss may contribute to neurodegeneration not only by influencing protein aggregation, but also through an immune mechanism. Therefore, we aimed to investigate whether promoting sleep as a therapeutic strategy could mitigate the damaging effects of chronic microglial reactivity that contribute to tau‐mediated neurodegeneration.

**Method:**

We used lemborexant, a dual orexin receptor antagonist that promotes sleep in both mice and humans. We orally gavaged P301S/APOE4 mice, a model of tauopathy with brain atrophy, and non‐tau depositing APOE4 knock‐in mice daily with 30mg/kg lemborexant or vehicle (n = 16‐20/genotype and treatment group) at one‐hour post‐dark onset. Mice were treated from 7.5 months (M), when tau‐mediated neuroinflammation is observed without overt neuronal loss in P301S/APOE4 mice, until 9.5M.

**Result:**

In P301S/APOE4 mice, lemborexant not only improved tau‐associated sleep loss, specifically non‐rapid eye movement sleep, but also dramatically reduced pathological tau deposition. Antagonizing orexin receptor signaling improved tau‐mediated neurodegeneration noted by a decrease in plasma neurofilament light chain levels, as well as brain atrophy compared to vehicle‐treated P301S/APOE4 controls. In support of these findings, lemborexant‐treated P301S/APOE4 mice displayed reduced microglial reactivity of disease‐associated microglia including immunostaining for CD68 and Clec7a compared to controls. Both astroglial and microglial APOE co‐localization were significantly reduced in lemborexant‐treated P301S/APOE4 mice, the latter of which is more commonly observed during elevated inflammatory and damaging conditions. Unbiased transcriptome profiling provided potential mechanistic insights into functional pathways influenced by lemborexant in P301S/APOE4 mice, including those regulating synaptic activity such as *Slc17a7, Shank1, Shank2*, which was further accompanied by reduced pre‐ and post‐synaptic loss.

**Conclusion:**

Our study provides novel therapeutic evidence that antagonizing the orexin signaling pathway using lemborexant is neuroprotective by restoring sleep deficits as well as limiting tau‐mediated neuronal and synaptic damage, potentially by suppressing chronic neuroinflammation.

